# The Directory of Second-Hand Booksellers and List of Public Libraries, British and Foreign

**Published:** 1891-06

**Authors:** 


*The Directory of Second-hand Booksellers and List of Public Libraries, British and Foreign.* Edited by JAMES CLEGG. Rochdale: JAMES CLEGG. 1891.

This book has been much improved since we noticed the previous issue, in September, 1889, although the deficiencies to which we then called attention have not been wholly supplied. Bristol, although identified from the beginning with the Free Library movement, does not appear in the list of Municipal Free Libraries. We should like to see an annual issue of the work, and the advertisements all kept together either at the beginning or the end.

